# Systematic profiling of mitochondria-related transcriptome in tumorigenesis, prognosis, and tumor immune microenvironment of intrahepatic cholangiocarcinoma: a multi-center cohort study

**DOI:** 10.3389/fgene.2024.1430885

**Published:** 2024-07-26

**Authors:** Bo Chen, Mengmeng Lu, Qiwen Chen, Enguang Zou, Zhiyuan Bo, Jiacheng Li, Rui Zhao, Jungang Zhao, Zhengping Yu, Gang Chen, Lijun Wu

**Affiliations:** ^1^ Department of Hepatobiliary Surgery, The First Affiliated Hospital of Wenzhou Medical University, Wenzhou, Zhejiang, China; ^2^ Zhejiang-Germany Interdisciplinary Joint Laboratory of Hepatobiliary-Pancreatic Tumor and Bioengineering, The First Affiliated Hospital of Wenzhou Medical University, Wenzhou, Zhejiang, China; ^3^ Department of Gastroenterology, The First Affiliated Hospital of Wenzhou Medical University, Wenzhou, Zhejiang, China

**Keywords:** intrahepatic cholangiocarcinoma, mitochondrial dysfunction, prognosis, tumor immune microenvironment, molecular subtype, multicentral study

## Abstract

**Background:**

Mitochondrial dysfunction has been shown to play a critical role in cancer biology. However, its involvement in intrahepatic cholangiocarcinoma (iCCA) remains significantly understudied.

**Methods:**

RNA sequencing data of 30 pairs of iCCA and paracancerous tissues were collected from the First Affiliated Hospital of Wenzhou Medical University (WMU). The WMU cohort (n = 30) was integrated with public TCGA (n = 30) and GSE107943 (n = 30) datasets to establish a multi-center iCCA cohort. We merged the TCGA and GSE107943 cohorts into an exploration cohort to develop a mitochondria signature for prognosis assessment, and utilized the WMU cohort for external validation. Gene Ontology (GO), Kyoto Encyclopedia of Genes and Genomes (KEGG), and Hallmarker analyses were used for functional interpretation of iCCA associated mitochondria-related genes (MRGs). In addition, unsupervised clustering was performed to identify mitochondria-based iCCA subtypes with the data of three institutions. Further investigations were conducted to examine the impact of mitochondrial dysfunction on drug responses, alteration of the tumor immune microenvironment, and immune responses.

**Results:**

Two hundred and sixty-three iCCA-related MRGs were identified to be related to fatty acid metabolism, oxidative phosphorylation, and apoptosis. Through univariate and multivariate Cox, and LASSO analyses, a mitochondria signature with five optimal MRGs was established to evaluate the prognosis of iCCA patients with the AUC values ranged from 0.785 to 0.928 in the exploration cohort. The signature also exhibited satisfactory performance in the WMU cohort with AUC values of 0.817–0.871, and was identified as an independent risk predictor in both cohorts. Additionally, we found that patients with higher mitochondria score with poor prognosis presented lower infiltration levels of CD4^+^ T-cell, NK cells, and monocytes, and demonstrated higher sensitivity to targeted therapies, including sorafenib. Furthermore, two distant mitochondria-based subtypes were determined, and subtype 2 was associated with shorter survival time and immunosuppressive tumor microenvironment. Finally, the differential protein expression of five key MRGs was verified by Immunohistochemistry.

**Conclusion:**

We found mitochondrial dysfunction modulates aberrant metabolism, oxidative stress, immune responses, apoptosis, and drug sensitivity in iCCA. A mitochondria signature and two mitochondria-based iCCA subtypes were identified for clinical risk stratification and immunophenotyping.

## Introduction

Intrahepatic cholangiocarcinoma (iCCA), which originates from the epithelial cells of intrahepatic second-order bile ducts, has witnessed an unexpected increase in its incidence and mortality worldwide over the past 10–30 years (Kilander et al., 2014; Rizvi et al., 2018; Sirica et al., 2019). Accounting for 10%–20% of primary hepatic malignancies, iCCA poses a significant challenge in early detection due to the absence of specific clinical manifestations, leading to a poor prognosis with a 5-year survival rate of approximately 30% (El-Diwany et al., 2019). Systemic chemotherapy becomes necessary for patients who are ineligible for surgery, but studies have demonstrated unsatisfactory outcomes and low overall survival (OS) rates (Endo et al., 2008; Kelley et al., 2020). Recent evidence has highlighted potential therapeutic roles of targeted therapies, such as erdafitinib targeting FGFR1-3, and immunotherapies, like Gem/Cis plus durvalumab, in the management of iCCA (Abou-Alfa et al., 2020; Kam et al., 2021). Nevertheless, the efficacy of these treatments remains limited without substantial improvement in prognosis (Lowery et al., 2019; Moris et al., 2023b). Therefore, further investigations are warranted to explore the underlying mechanisms of iCCA progression. These efforts are crucial for identifying novel biomarkers for early detection, developing prognostic signatures, and identifying potentially effective targeted treatment strategies.

Mitochondria, as the powerhouse of eukaryotic cells, play a critical role in generating adenosine triphosphate (ATP) for cellular metabolism, which is essential for cell survival and proper functioning. Additionally, mitochondria are extensively involved in various biological processes including apoptosis, calcium homeostasis, and oxidative stress response. Consequently, any genetic mutations or abnormalities that interfere with mitochondrial function can significantly impact cellular metabolism and promote tumorigenesis and recurrence (Greaves et al., 2012; Tan et al., 2021). Metabolic reprogramming, a pivotal characteristic of cancer cells, involves disruptions in metabolic pathways, resulting in alterations in nutrient uptake and utilization. Several genes associated with mitochondrial function are involved in this process, including those responsible for mitochondrial DNA damage, impairment of the respiratory chain, changes in mitochondrial membrane potential, and other factors affecting mitochondrial function (Hanahan and Weinberg, 2011).

Numerous studies have demonstrated that various tumors undergo metabolic reprogramming, leading to dysregulation in the expression of mitochondria-related genes (MRGs) responsible for encoding proteins such as mitochondrial uncoupling protein 2 (UCP2) and mitochondrial calcium uniporter (MCU) (Raho et al., 2020; Wang et al., 2022). It is suggested that mitochondrial dysfunction is closely linked to the initiation and progression of cancer. Additionally, the presence of mitochondrial dysfunction and metabolic reprogramming can lead to variations in the effectiveness of immunotherapy due to intra-tumor heterogeneity, primarily influenced by changes in the tumor immune microenvironment (TIME) (Yoshida, 2015). Investigating key regulatory factors and inhibitory elements of treatment resistance within the TIME is essential for advancing new therapeutic strategies (Ashrafizadeh et al., 2024; Dai et al., 2024). Previous research has indicated the crucial involvement of mitochondrial oxidative metabolism in the pathogenesis of cholangiocarcinoma (Morin et al., 2014; Raggi et al., 2021). However, the roles of mitochondrial dysfunction in the occurrence and progression of iCCA as well as in altering the TIME remain poorly understood. Further exploration may facilitate the identification of potential therapeutic targets.

In the present study, we established a transcriptome cohort of iCCA from the First Affiliated Hospital of Wenzhou Medical University (WMU), integrating TCGA and GEO datasets, to elucidate the exact relationship of mitochondria dysfunction with iCCA. Moreover, a mitochondria signature and two mitochondria-based iCCA subtypes were identified based on the multi-center cohort for clinical risk stratification and immunophenotyping.

## Methods and materials

### Patient cohorts

Patients with iCCA who underwent curative resection from the First Affiliated Hospital of Wenzhou Medical University were retrospectively recruited from October 2017 to November 2019. The inclusion criteria were as follows (Rizvi et al., 2018): pathologically confirmed iCCA (Sirica et al., 2019) undergoing curative liver resection (Kilander et al., 2014); availability of non-degraded tissue specimens; and (El-Diwany et al., 2019) a performance status (PS) score of 0–2. The exclusion criteria were as follows (Rizvi et al., 2018): presence of other malignancies (Sirica et al., 2019); incomplete clinical information; and (Kilander et al., 2014) OS of less than 1 month due to postoperative complications. Finally, thirty pairs of iCCA and paracancer tissues were obtained with informed consent form patients. Paracancerous tissue refers to the normal liver tissue surrounding iCCA, typically sampled 2–3 cm from the tumor margin, adjusted based on the specific tumor size. It is determined by specialized pathologists through rigorous pathological examination to ensure the absence of cancer cell infiltration. All patients in the WMU cohort were diagnosed with iCCA by two independent pathologists, and detailed demographic data and tumor characteristics were collected. All patients underwent curative resection. For those with vascular invasion, our team conducted extensive liver resection to ensure comprehensive excision of all affected tissues, including involved vessels. Vascular reconstruction may be required in certain cases. OS refers to the interval between the data of hepatectomy and the date of death from any cause. This study was approved by the ethics committee of The First Affiliated Hospital of Wenzhou Medical University (IRB approval number: 2020-074) and adhered to the Declaration of Helsinki.

Two other publicly available cohorts with RNA sequencing data and follow-up information, TCGA-CHOL from the TCGA database and GSE107943 from the GEO database, were also included into this study. The TCGA-CHOL cohort consists of 8 normal and 30 iCCA tissues, and GSE107943 cohort contains 27 normal and 30 iCCA tissues, respectively. For subsequent research on prognostic models, we merged the TCGA and GSE107943 cohorts into an exploration cohort by eliminating batch effects, while the WMU cohort served as an external validation cohort.

### Tumor samples and RNA-sequencing

Total RNA was extracted from the frozen 30 pairs of iCCA and paracancer tissues using the RNAeasyTM Animal RNA Isolation Kit with Spin Column (Beyotime) in line with the manufacturer’s protocol. RNA-sequencing was performed by BGI China. Following the isolation of the RNA, the integrity of each RNA sample was verified by Bioanalyser (Agilent Technologies) according to the protocol, and only those RNAs that passed this quality control were included to transcriptome sequencing analysis. After enrichment, purification, fragmentation, reverse transcription and specific amplification, the RNA yielded single-stranded circular DNA libraries, which then underwent sequencing on the Illumina HiSeq platform. We filtered out reads containing adapters, low quality reads, and reads with an N content greater than 5% using SOAPnuke (v1.4.0), followed by alignment of the clean reads to the reference genome using Bowtie2 (v2.2.5) (Langmead and Salzberg, 2012). Gene and transcript expression levels were quantified with RSEM (Li and Dewey, 2011). Utilization of metrics such as sequence coverage, GC content distribution, and error rate analysis serves as critical quality control standards for assessing the quality of sequencing data.

### ICCA-related MRGs and functional interpretation

Mitochondria-related genes (MRGs) were gathered from MitoCarta v3.0 and Mitominer v4.0 databases (Smith and Robinson, 2019; Rath et al., 2021). A total of 1,136 genes were sourced from MitoCarta v3.0, 1,626 genes from Mitominer v4.0, resulting in a union of 1,718 MRGs (Supplementary Table S1). The transcriptome data from three databases were converted to FPKM (Fragments Per Kilobase of transcript per Million mapped reads) values and normalized to log2 (FPKM + 1) format. Then, the wilcoxon signed-rank test was performed to identify differentially expressed MRGs in the iCCA and paracancer tissues with the LIMMA package in the TCGA and GSE107943 datasets, respectively. We strictly set the screening criteria as |log2 fold change| > 2 and *P*-value < 0.05. Subsequently, two Venn plots were drawn to screen valuable iCCA-related MRGs that were upregulated or downregulated in both the TCGA and GSE107943 datasets.

Furthermore, to interrogate the latent pathophysiological processions and mechanisms deep inside the mitochondrial dysfunction, we undertook functional enrichment analyses for iCCA-related MRGs, including Gene Ontology (GO), Kyoto Encyclopedia of Genes and Genomes (KEGG) pathway, and Hallermaker pathway analyses. We identified results with an adjusted *P*-value < 0.05, and visualized the top 10 significant terms.

### A distinct mitochondria signature for prognosis evaluation

First, after intersecting the two RNA-seq datasets from TCGA and GSE107943, we retained only the expression data of the common genes. Then, we integrated the gene expression profiles of iCCA patients from the TCGA and GSE107943 cohorts and performed batch effect correction using the Combat function within the sva R package (Johnson et al., 2007), resulting in an RNA-seq exploratory cohort containing 60 tumor patients. Univariate Cox regression analysis was implemented to screen MRGs related to OS with a threshold of *P* < 0.05 using survival R package. To address the issue of redundant genes that may affect the precision of the signature, we further employed LASSO analysis with the glmnet R package. This approach helps to identify a subset of relevant genes by performing feature selection and regularization to achieve better predictive performance. Following that, significant OS-related MRGs from the LASSO analysis were incorporated into the multivariate cox regression to construct the mitochondria signature according to the minimum akaike information criterion (AIC) method with the survival R package. The mitochondria score of each patient was calculated from the expression of valuable MRGs with standardized regression coefficients in the multivariate analysis. The calculation formula was as follow: mitochondria score = $\sum_{i=1}^{n}\beta i*\text{Gi}$ . Here, “βi” is the estimated regression coefficient of the gene from the multivariate Cox proportional hazards regression analysis, and “Gi” is the expression of MRGs.

Based on the median mitochondria score, iCCA patients were divided into high- and low-risk groups. Kaplan-Meier (KM) survival analysis with log-rank test was conducted to compare the survival status between these two groups with survminer R package. Additionally, we employed time-dependent receiver operating characteristic (ROC) curves and corresponding area under the curve (AUC) values to evaluate the prognostic performance of the mitochondria signature at 12, 24, and 36-month time intervals with the survivalROC R package.

Considering clinical characteristics could also contribute to differences in OS, we incorporated the mitochondria score along with clinical variables into both univariate and multivariate cox regression models, including age, gender, CA199, grade stage, AJCC stage, and vascular invasion. Vascular invasion was defined as the invasion of the portal vein, hepatic artery, or hepatic veins. This enabled us to validate the mitochondria signature as an independent risk factor while comparing its predictive performance against established clinical indices.

### Tumor immune microenvironment assessment

We employed the ESTIMATE algorithm to derive three types of tumor immune microenvironment (TIME)-related scores, thereby ascertaining the relative abundances of stromal and immune cellular constituents within iCCA patient samples.

### Immune cell infiltration

The fractions of 22 infiltrating immune cell types were determined with the CIBERSORT package, and only iCCA patients with significant results (*P* < 0.05) were obtained. To comprehensively analyze the immune cells from various angles, we also utilized two algorithms, namely, quanTIseq and TIMER (Finotello et al., 2019; Li et al., 2016), which were able to accurately calculate the main ten and six immune cell components in the microenvironment, respectively.

### Immune-related genes

Twenty-nine immune-associated gene sets were calculated by single-sample Gene Set Enrichment Analysis (ssGSEA) with “GSEABase” package. In addition, the expression of 60 immune checkpoint genes and 150 immunomodulatory genes were obtained to assess the immune response.

### Drug sensitivity analysis

With the utilization of the pRRophetic package, drug response models combining baseline gene expression and drug half-maximal inhibitory concentration (IC50) in diverse cell lines were derived (Geeleher et al., 2014). The *t*-test was employed to compare differences in IC50 of common chemotherapeutic agents between the different groups. The outcomes with *P* < 0.01 were deemed statistically significant.

### External verification

Validating the predictive accuracy of a model in an external cohort is of utmost importance. The expression data for constituent genes were extracted from the WMU cohort of 30 iCCA patients and input into the equations for computing mitochondria scores. Median mitochondria score was implemented to stratify patients into high- and low-risk groups. The predictive capacity of the mitochondria signature was subsequently verified through ROC and K-M survival curves. Moreover, univariate and multivariate Cox regression analyses were employed to assess the presence of independent risk factors in the WMU cohort.

The expression level of crucial MRGs in the signature were derived from the WMU cohort, and their differential expression was verified in 30 paired paracancer and cancer tissues.

### Identification of mitochondria-based iCCA subtypes via unsupervised clustering

Defining novel molecular subtypes of cancers may enable precise diagnosis, prognosis evaluation, tailored treatment strategies, and individualized patient care. To derive a robust consensus, we aggregated the WMU, TCGA and GSE107943 cohorts, yielding an integrated cohort comprising 90 iCCA patients for discovery-based analysis. A K-medoids clustering algorithm, called partitioning around medoids (PAM) was employed to evaluate the similarity among iCCA patients based on MRGs with ConsensusClusterPlus package (Wilkerson and Hayes, 2010), to identify potential mitochondria-based iCCA subtypes for clinical application. The elbow method and gap statistic were performed to determine the optimal number of clusters, and principal component analysis (PCA) was undertaken to substantiate the veracity of clustering outcomes. Thereafter, we compared survival status of different subtypes using the KM survival curve to probe the relationship between the functional status of mitochondria and iCCA prognosis. In addition, following the derivation of the molecular subtypes with a pronounced prognosis discrepancy, we interrogated distinctions in their corresponding immune microenvironments, immunological cellular components, immune regulatory genes, and immune checkpoint genes to probe for interconnections with mitochondrial dysfunction.

### Immunohistochemistry (IHC)

After embedding, tumor and paracancer tissues were sectioned into 4 μm paraffin slices, followed by deparaffinization and hydration. Then, paraffin sections were subjected to antigen retrieval using citrate, and endogenous peroxidase activity was blocked with 3% H2O2, followed by blocking with 5% BSA. Sections were then cultured with primary antibodies overnight at 4°C. The primary antibodies utilized in this study include: ANXA1 (21990-1-AP, Proteintech), BCL2 (AF6139, Affinity), GPT2 (16757-1-AP, Proteintech), SNPH (13646-1-AP, Proteintech), and TUSC3 (16039-1-AP, Proteintech). The following day, after incubation with secondary antibodies, sections were dyed with 3,3’-diaminobenzidine (DAB). Counterstaining with hematoxylin was performed, followed by air-drying and mounting with neutral resin. Finally, all slides were observed and photographed under a microscope. The IHC Profiler plugin of Image J was utilized for quantitative analysis.

### Statistical analysis

All statistical analyses were performed in R-Studio software (version 4.2.0). The intergroup differences were analyzed using Wilcoxon signed-rank test, except for the drug sensitivity analysis where a *t*-test was employed. Univariate and multivariate Cox analyses were performed for selection of survival-related factors. A *P*-value < 0.05 was deemed statistically significant with the exception of drug sensitivity analysis for which a *P*-value < 0.01 was considered the threshold.

## Results

### ICCA patient characteristics

The workflow of this study was shown in Supplementary Figure S1. A total of 90 patients with iCCA from three institutions were included in the final cohort (Table 1). The mean patient age was 62.90 ± 13.54, 65.60 ± 8.74, and 62.47 ± 9.55, and median CA199 level was 34.00 (125.75), 28.18 (146.02), and 42.60 (217.7) in the TCGA, GSE107943, and WMU cohorts, respectively. Of the patients, 14 (46.67%), 24 (80.00%), and 11 (36.67%) were male, 14 (46.67%), 21 (70.00%), and 19 (63.33%) presented with I-II histological stage, and 27 (90.00%), 21 (70.00%), and 26 (86.67%) had a TNM stage of I-II.

**TABLE 1:** Clinical characteristics of intrahepatic cholangiocarcinoma (iCCA) patients in the multi-cohort.

	Exploration cohort		External validation cohort
Characteristic	TCGA cohort	GSE107943	WMU cohort
Age, year	62.90 ± 13.54	65.60 ± 8.74	62.47 ± 9.55
Gender			
Female	16 (53.33%)	6 (20.00%)	19 (63.33%)
Male	14 (46.67%)	24 (80.00%)	11 (36.67%)
BMI, kg/m2	27.73 ± 5.13	—	22.61 ± 3.07
Hepatitis B	—	4 (13.33%)	13 (43.33%)
CEA, ng/mL	—	2.10 (2.68)	1.95 (2.85)
CA199, U/mL	34.00 (125.75)	28.18 (146.02)	42.60 (217.7)
Grade			
I-II	14 (46.67%)	21 (70.00%)	19 (63.33%)
III-IV	16 (53.33%)	7 (23.33%)	10 (33.33%)
Unknow	0 (0.00%)	2 (6.67%)	1 (3.33%)
Tumor size, mm	—	5.00 (3.25)	4.10 (4.20)
TNM stage			
I-II	27 (90.00%)	21 (70.00%)	26 (86.67%)
III-IV	3 (10.00%)	9 (30.00%)	4 (13.33%)
Vascular invasion			
No	25 (83.33%)	18 (60.00%)	22 (73.33%)
Yes	4 (13.33%)	12 (40.00%)	8 (26.67%)
Unknow	1 (3.33%)	0 (0.00%)	0 (0.00%)

### Overview of MRGs in normal and iCCA tissues

**FIGURE 1:**
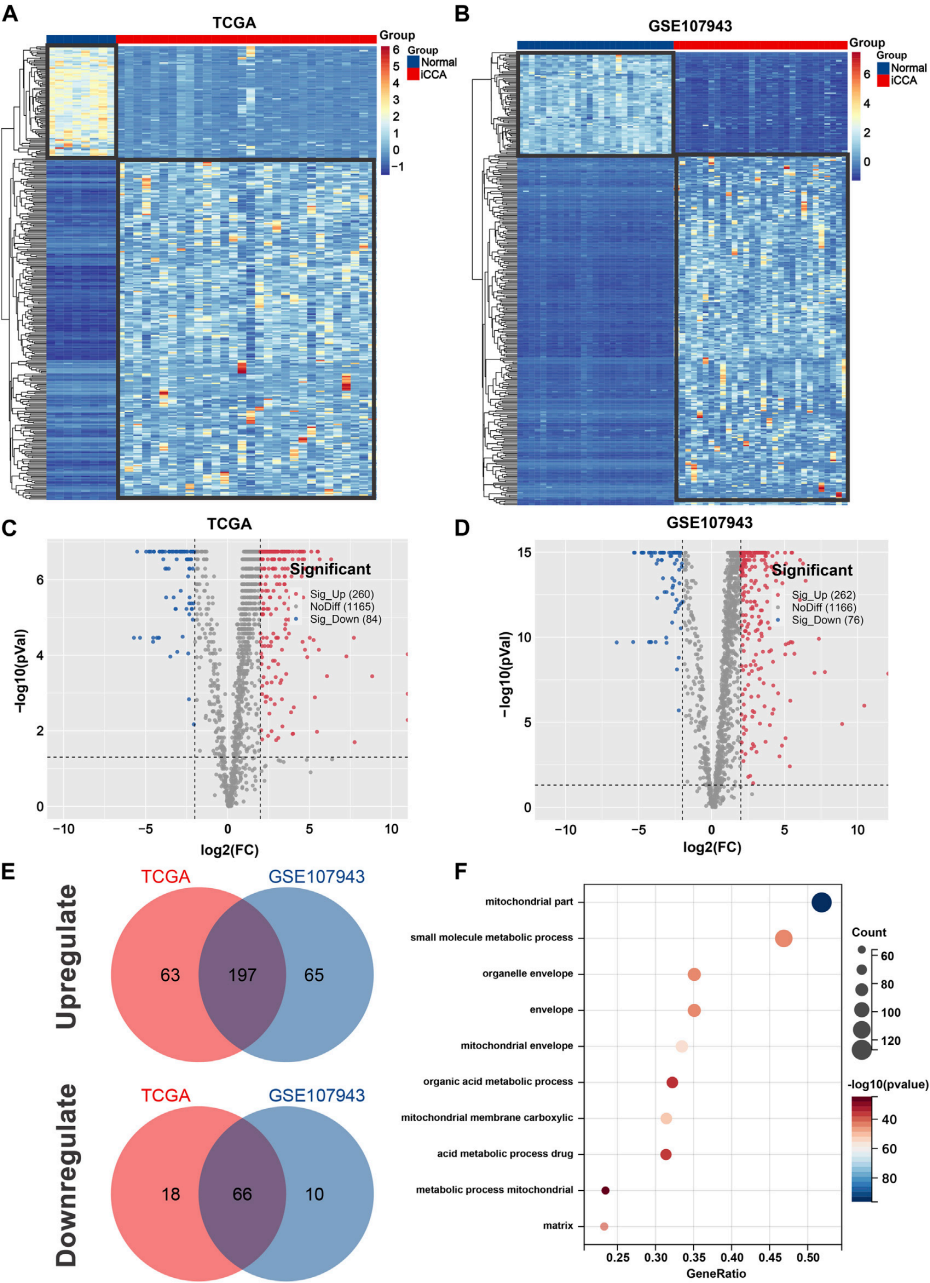
Identification of iCCA-related MRGs in the TCGA and GSE107943 datasets. Heatmap **(A)** and volcano plot **(C)** of 344 differentially expressed MRGs in the TCGA dataset. Heatmap **(B)** and volcano plot **(D)** of 338 differentially expressed MRGs in the GSE107943 dataset. **(E)** Venn diagrams of 197 commonly unregulated and 66 commonly downregulated MRGs in both datasets. **(F)** Bar graph of the 10 most significant terms from GO enrichment analysis. iCCA, Intrahepatic cholangiocarcinoma; MRGs, mitochondria-related genes; GO, Gene ontology.

After screen, 344 iCCA-related MRGs were identified in the TCGA cohort, with 260 upregulated and 84 downregulated genes (Figures 1A, C), and 338 iCCA-related MRGs were revealed in the GSE107943 cohort, with 262 upregulated and 76 downregulated genes (Figures 1B, D). Notably, 197 commonly upregulated MRGs and 66 commonly downregulated MRGs were mined, as shown in Venn diagrams (Figure 1E).

**FIGURE 2:**
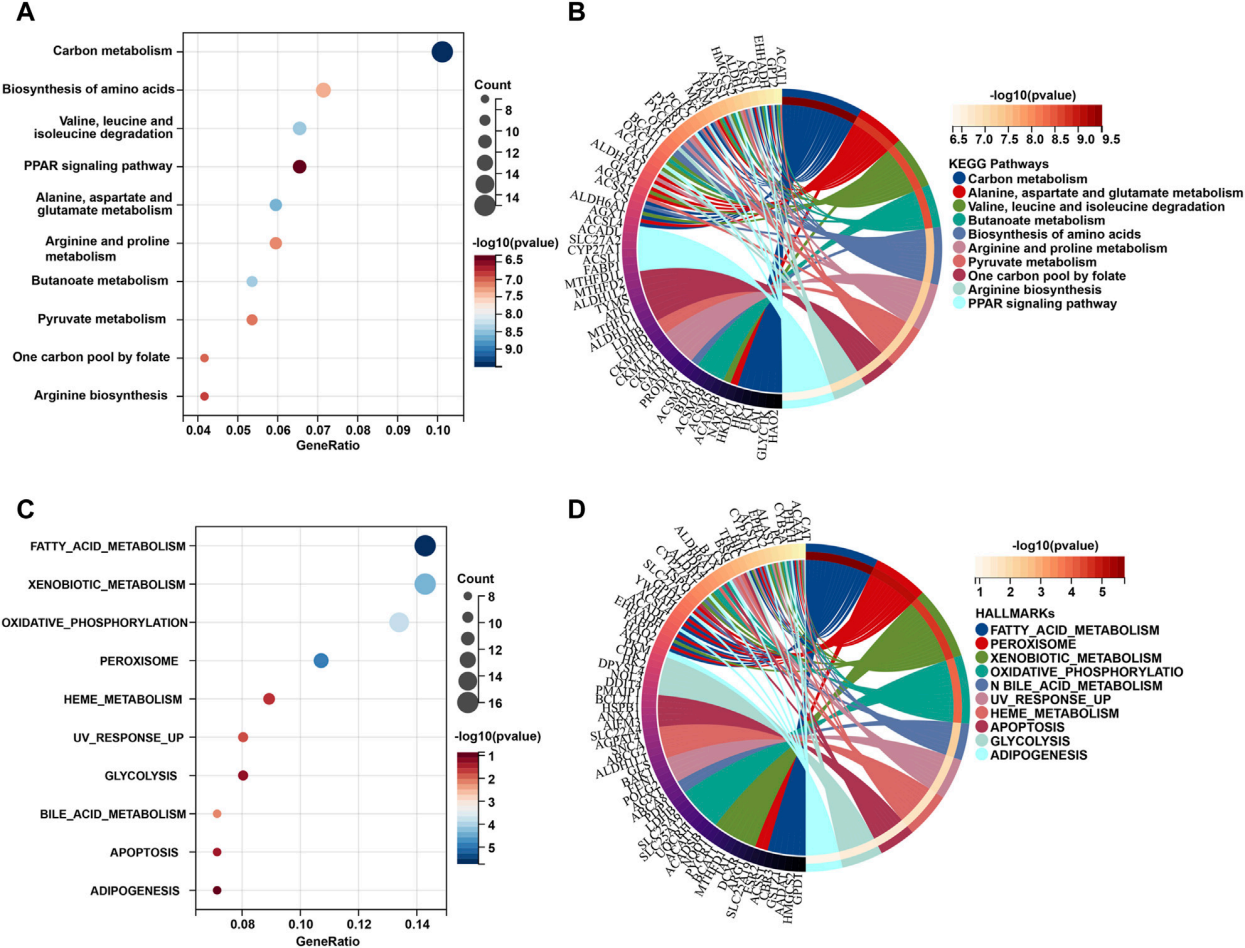
KEGG and Hallmark pathway functional interpretation of iCCA-related MRGs. Bubble plot **(A)** and circle diagram **(B)** of the 10 most significant terms form KEGG pathway analysis. Bubble plot **(C)** and circle diagram **(D)** of the 10 most significant terms form Hallmark pathway analysis. MRGs: mitochondria-related genes.

To gain a better understanding of how these robust survival-related MRGs may drive iCCA development, functional annotation was performed. We found that the most significant GO enriched terms were mitochondrial part, small molecule metabolic process, mitochondrial envelope, mitochondrial membrane carboxylic, and acid metabolic process drug (Figure 1F). Furthermore, KEGG pathway analysis revealed that MRGs were predominantly associated with carbon metabolism, biosynthesis of amino acids, valine, leucine and isoleucine degradation, PPAR signaling pathway, alanine, aspartate and glutamate metabolism, arginine and proline metabolism, butanoate metabolism, one carbon pool by folate, and arginine biosynthesis (Figures 2A, B), the aberrations of which are pivotal drivers in carcinogenesis and cancer progression. Additionally, Hallmarks pathway analysis showed that iCCA-related MRGs were mainly enriched in fatty acid metabolism, xenobiotic metabolism, oxidative phosphorylation, glycolysis, bile acid metabolism, apoptosis, and adipogenesis (Figures 2C, D). These results indicated that mitochondrial dysfunction may serve as an important intermediary in iCCA tumorigenesis via metabolic abnormalities and oxidative stress.

### A distinct mitochondria signature for prognosis evaluation

**FIGURE 3:**
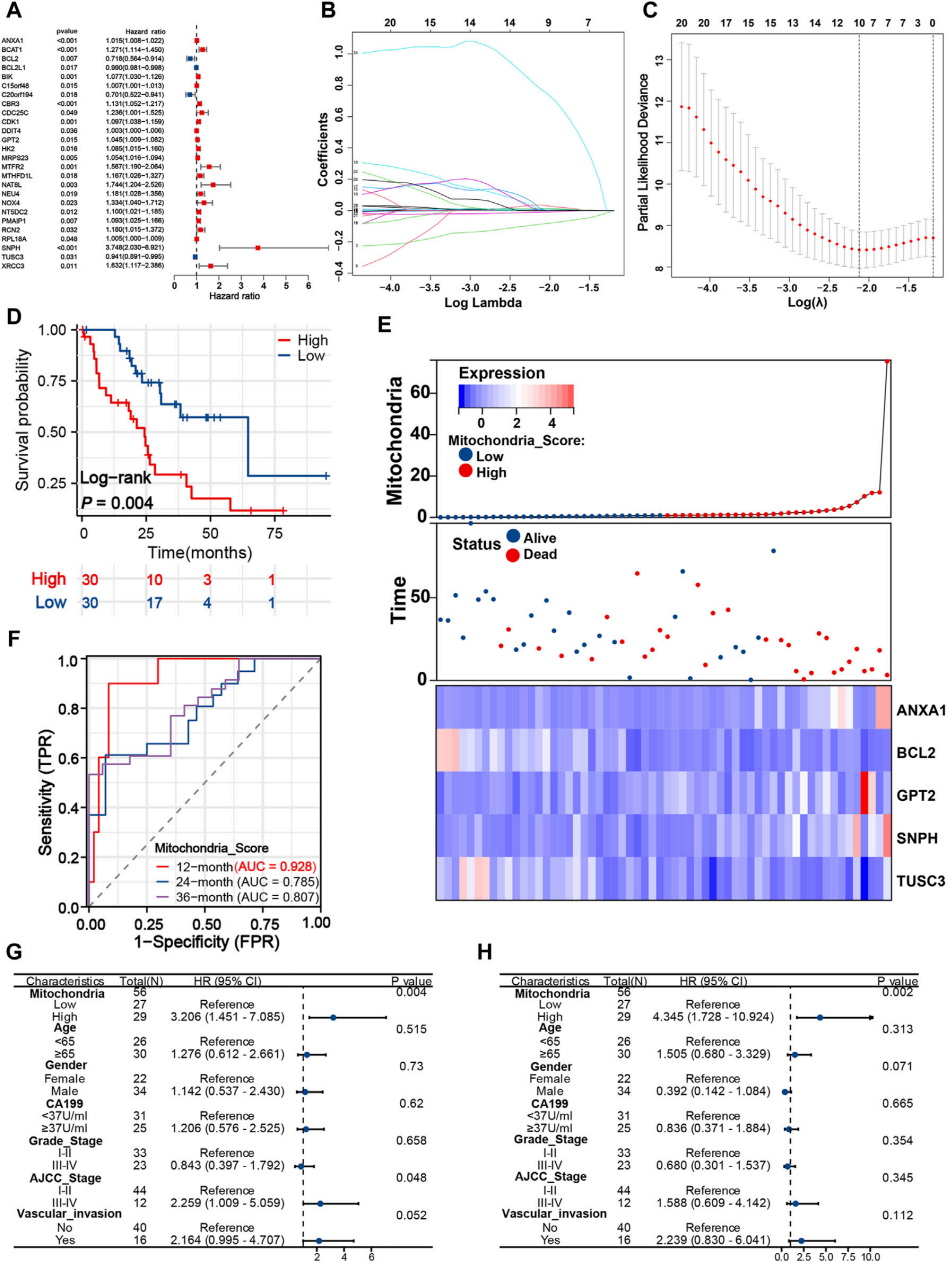
Developing a distinct mitochondria signature for prognosis evaluation. **(A)** Twenty-Six survival-related MRGs identified from univariate Cox analysis. LASSO coefficient **(B)** and deviance profiles **(C)** of survival-related MRGs. **(D)** K-M survival curves of patients in the different risk groups based on the mitochondria signature. **(E)** Heatmap, survival status plot and risk score plot of the mitochondria signature. **(F)** Time-dependent ROC curves of the signature at 12, 24, and 36 months. Univariate **(G)** and multivariate Cox analyses **(H)** of mitochondria signature and other clinical predictors. MRGs, mitochondria-related genes; K-M, Kaplan-Meier; ROC, Receiver operating characteristic.

Assessment of the prognosis of iCCA is a crucial task, yet effective prognostic methods are still lacking. Further research and development of more reliable and accurate prognostic tools for iCCA are necessary to guide clinical treatment and prognosis monitoring. As shown in Figure 3A, univariate Cox regression analysis determined 26 survival-related MRGs for iCCA patients in the exploration cohort, among which SNPH gene expression was found to be most significantly correlated with poor prognosis (HR = 3.748, 95% CI = 2.030–6.921, *P* < 0.001). Then, LASSO analysis was employed, and 10 vital candidate genes were retained (Figures 3B, C). On top of that, five robust MRGs were computed applicable for the signature construction with multivariate Cox regression analysis, and the mitochondria score of each patient was obtained: 0.009*ANXA1 expression–0.214*BCL2 expression + 0.037*GPT2 expression + 1.324*SNPH expression - 0.041*TUSC3 expression. Subsequently, patients from the exploration cohort were split into high- (n = 30) and low-risk (n = 30) groups with the median mitochondria score. Patients with a high mitochondrial score display significantly higher mortality rate and shorter survival time (Figure 3D). The results of time-dependent ROC curves showed that the mitochondria signature achieved the sufficient AUC of 0.928 at 12 months, and 0.785, and 0.807 at 24- and 36 months, respectively (Figure 3F). In addition, we visualized the relationship between mitochondrial score distribution, survival status distribution, and expression of vital genes (Figure 3E).

To gain a deeper insight into the clinical applicability and prognostic value of the mitochondrial signature, univariate and multivariate Cox analyses were implemented. Univariate analysis revealed that mitochondria score (HR = 3.206, 95% CI = 1.451–7.085, *P* = 0.004) and AJCC stage (HR = 2.259, 95% CI = 1.009–5.059, *P* = 0.048) were significantly associated with poor prognosis, while the remaining variables did not reach statistical significance in the exploration cohort (Figure 3G). On multivariable analysis of these clinically common prognostic predictors, only mitochondria score (HR = 4.345, 95% CI = 1.728–10.924, *P* = 0.002) was identified as an independent risk factor (Figure 3H).

### Immune characteristics and GSEA between different risk groups

**FIGURE 4:**
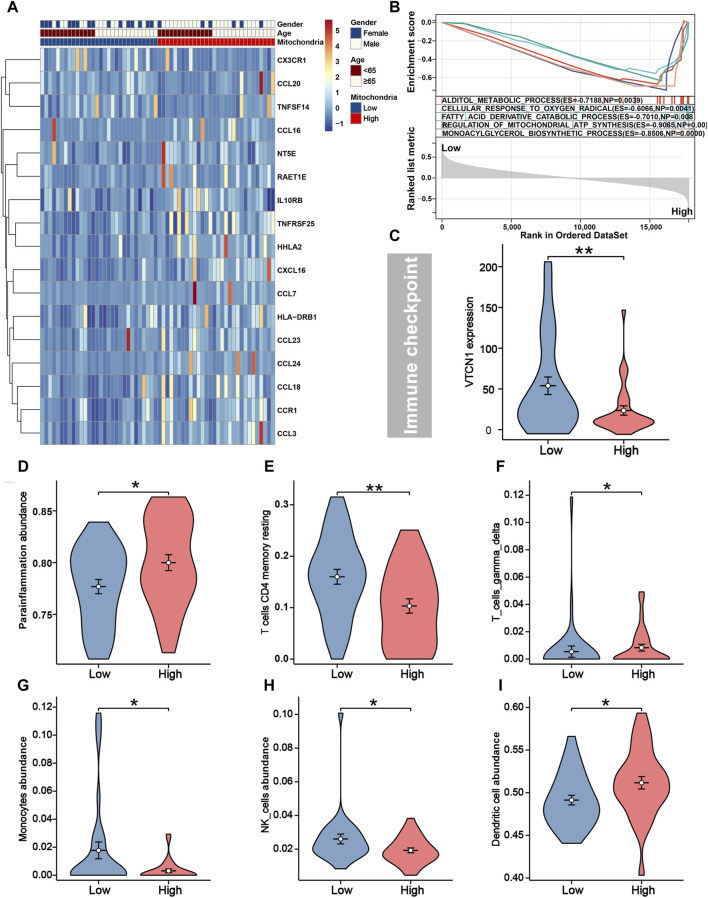
Immune characteristics and GSEA between different risk groups. **(A)** Seventeen immune genes were differentially expressed between different risk groups. **(B)** The top five biological activities were enriched in the high-risk group from GSEA analysis. **(C)** Immune checkpoint gene VTCN1 was higher expressed in patients with low-risk. The infiltration levels of parainflammation **(D)**, T-cell CD4 memory **(E)**, T-cell gamma delta **(F)**, monocytes **(G)**, NK cells **(H)**, and dendritic **(I)** were distinguishable between different risk groups.

As shown in Figure 4A, apart from CX3CR1, CCL20, and TNFSF14, the remaining immune genes demonstrated high expression in the high-risk group. The high expression of these immune genes may be attributed to the tumor cells’ ability to evade the immune system. Tumor cells may upregulate the expression of certain immune molecules as a means to escape immune surveillance and destruction. Generally, the majority of immune checkpoint genes and infiltrating immune cells did not differ significantly between groups. However, the expression level of VTCN1 was higher in the low-risk group (Figure 4C), which implied that it may serve as a potential immunotherapeutic target. We found iCCA patients in the low-risk group tended to present significantly higher infiltration levels of T-cell CD4 memory resting, monocytes, and NK cells, and lower infiltration levels of parainflammation, T-cell gamma delta, and dendritic cells (Figures 4D–I). In addition, we employed GSEA analysis to elucidate the potential biological activities involved in the heterogeneity behind different mitochondria scores. The results suggested that alditol metabolic process (ES = −0.7188, *P* = 0.0039), cellular response to oxygen radical (ES = −0.6066, *P* = 0.0041), fatty acid derivative catabolic process (ES = −0.7010, *P* = 0.008), regulation of mitochondrial ATP synthesis (ES = −0.9065, *P* = 0.000) and monoacylglycerol biosynthetic process (ES = −0.8506, *P* = 0.000) were significantly enriched in high-risk group with unsatisfactory survival (Figure 4B).

### Chemotherapeutic and targeted drug sensitivities evaluation

**FIGURE 5:**
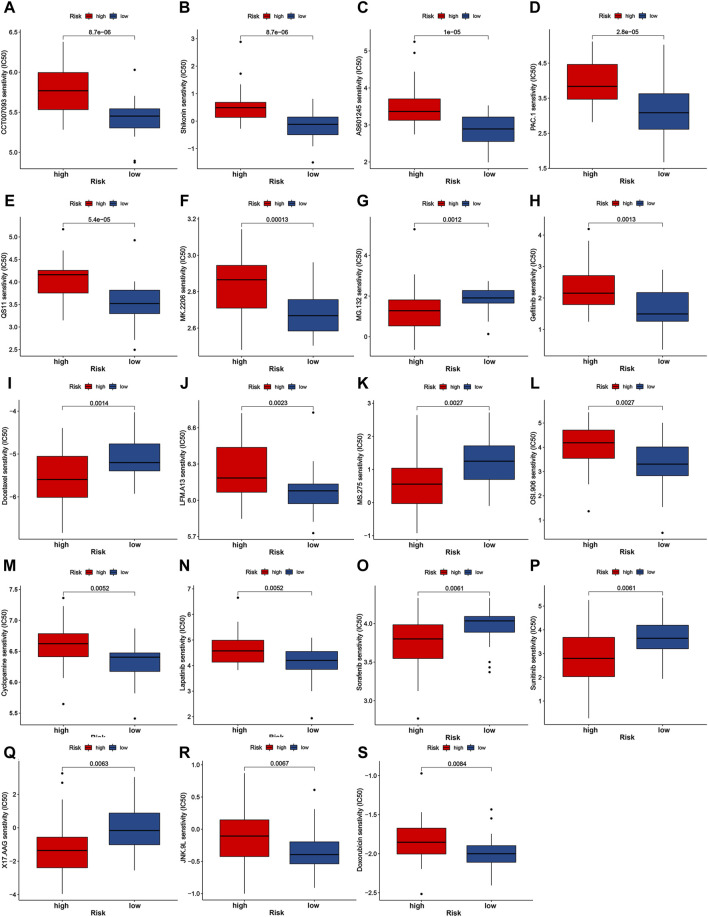
Drug sensitivity analysis of nineteen chemotherapeutic and targeted molecule compounds **(A–S)**.

We further evaluate the chemotherapeutic and targeted treatment sensitivity of iCCA patients with different mitochondria score (Figures 5A–S). The low-risk group exhibited higher IC50 for MG.132, Docetaxel, MS.275, Sorafenib, Sunitinib, and X17.AAG, implying that patients with higher mitochondria score may gain advantages from these precision medicines. Moreover, remaining molecular drugs has higher IC50 in high-risk group, including CCT007093, Shikonin, AS601245, PAC.1, QS11, MK.2206, Gefitinib, LFM.A13, OSI.906, Cyclopamine, Lapatinib, JNK.9L and Doxorubicin. These data imply that aberrations in mitochondrial function may be linked to pharmacologic sensitivity, indicating mitochondria score could emerge as a critical instrument for guiding treatment protocols.

### Predictive performance of mitochondria signature in the WMU cohort

**FIGURE 6:**
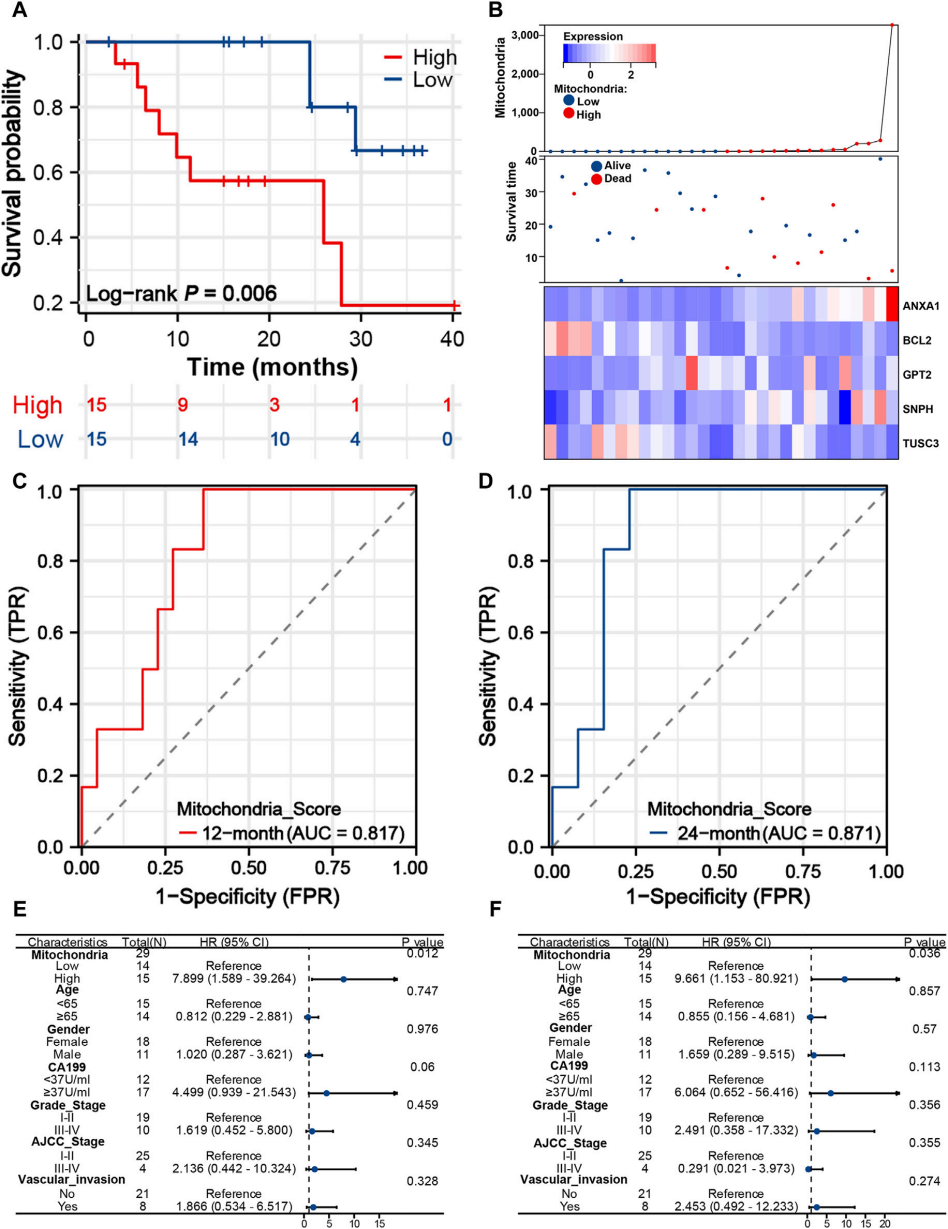
Predictive performance of mitochondria signature in the WMU cohort. **(A)** K-M survival curves of iCCA patients with different mitochondria score in the WMU cohort. **(B)** Heatmap, survival status plot and risk score plot of the mitochondria signature. Time-dependent ROC curves of the signature at 12 **(C)** and 24 **(D)** months. Univariate **(E)** and multivariate Cox analyses **(F)** of mitochondria signature and other clinical predictors. iCCA, Intrahepatic cholangiocarcinoma; K–M, Kaplan-Meier; ROC, Receiver operating characteristic.

The mitochondria scores of 30 iCCA patients were calculated according to the above formula in the WMU cohort. Also, patients were divided into low- and high-risk groups (Figure 6A), and patients with lower mitochondria score showed a significant survival advantage (*P* = 0.006). The AUC values of mitochondria signature were 0.817 and 0.871 at 12 and 24 months (Figures 6C, D), which suggested that the signature may also be used as a precise clinical prediction tool in the external cohort. We plotted the distribution of survival time and mitochondria score to clearly visualize differences in the prognosis and crucial gene expression (Figure 6B). Notably, univariate and multivariate analysis explored that mitochondria score was the sole independent risk factor in comparison to other commonly used clinical prognostic indicators (Figures 6E, F). This demonstrates that mitochondria score is a widely applicable biomarker in clinical settings, possessing both biological significance and high precision.

### Mitochondria-based iCCA subtypes associated with TIME and immune response

**FIGURE 7:**
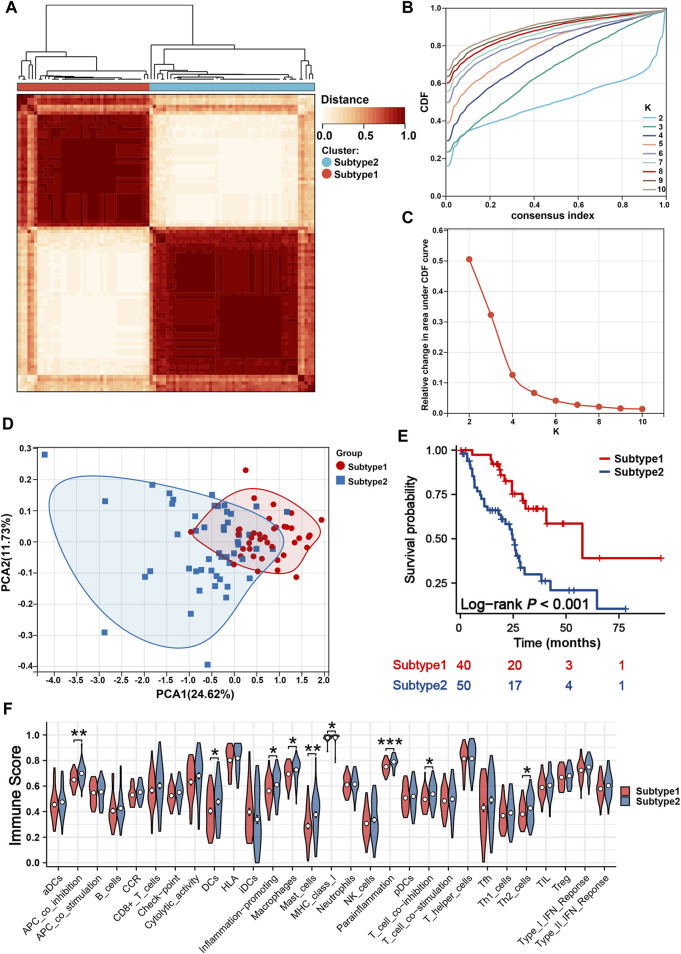
Identification of two mitochondria-based iCCA subtypes. **(A)** Consensus matrix heatmap defined two subtypes. **(B)** Cumulative Distribution Curve. **(C)** Area under the cumulative distribution curve. **(D)** PCA verified the two subtypes had significant differences. **(E)** K-M survival curves of iCCA patients with different subtypes. **(F)** The enrichment levels of 29 immune-related gene sets of iCCA patients in different subtypes. iCCA, Intrahepatic cholangiocarcinoma; PCA, Principal component analysis; K-M, Kaplan-Meier.

**FIGURE 8:**
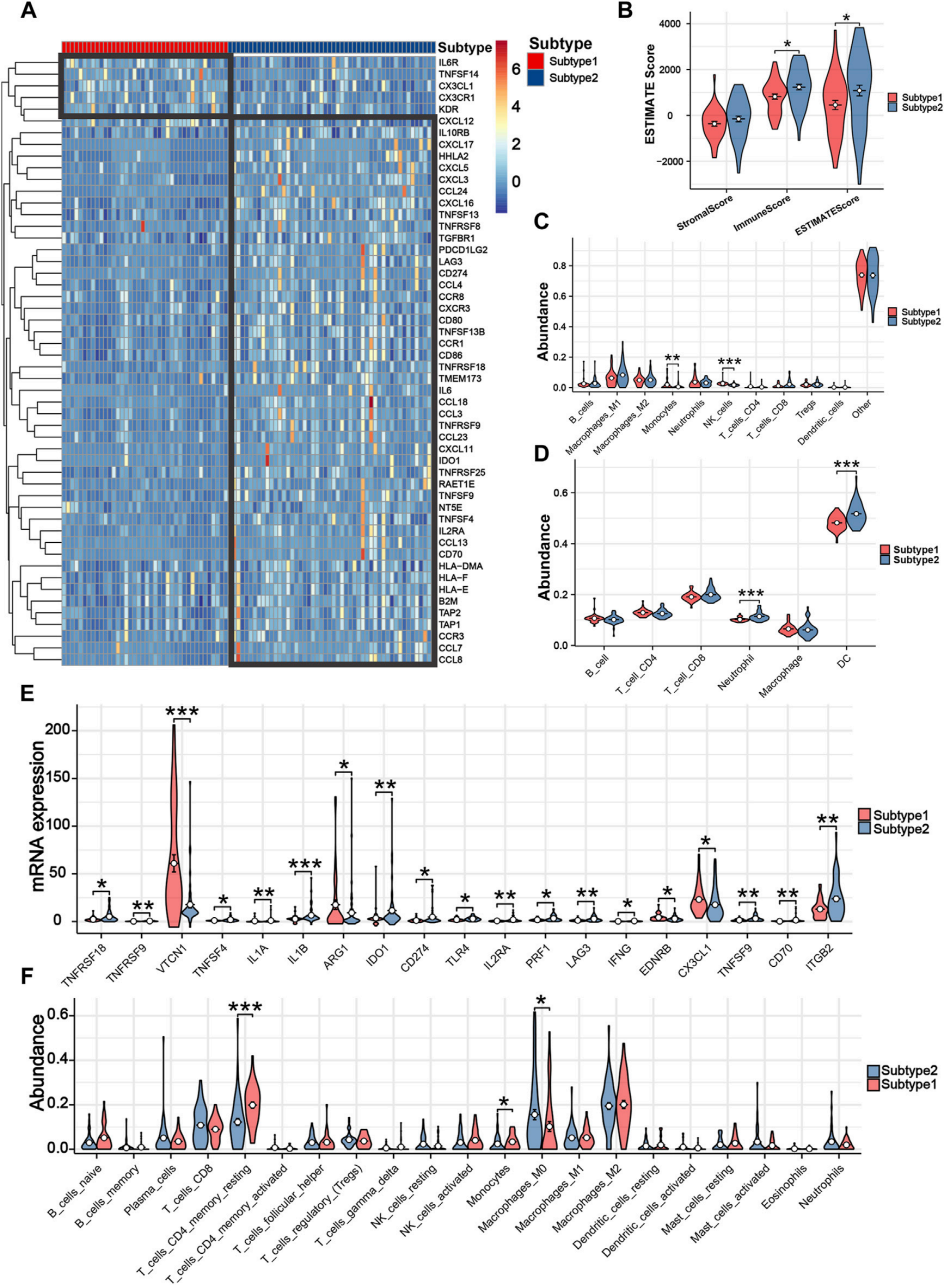
Tumor immune microenvironment and immune response assessment between different subtypes. **(A)** The differences among 52 immune genes of iCCA patients in different subtypes. **(B)** Three TIME-related scores between patients with different subtypes. **(C)** The infiltration levels of 11 immune cells based on quanTIseq algorithm. **(D)** The infiltration levels of six immune cells based on TIMER algorithm. **(E)** The differences among 19 immune checkpoint genes of iCCA patients in different subtypes. **(F)** The infiltration levels of 21 immune cells based on CIBESORT algorithm.

Two iCCA subtypes were identified from the unsupervised clustering (Figures 7A–C): subtype 1 (n = 40) and subtype 2 (n = 50). PCA verified that the two subtypes had significant differences and were almost completely distinguishable (Figure 7D). We observed significant differences in survival between the two subtypes, with patients in subtype 1 exhibiting noticeably longer OS (Figure 7E). The results of 29 immune-associated gene sets based on ssGSEA are shown in Figure 7F. We found APC co-inhibition, DCs, inflammation promoting, Macrophages, Mast cells, MHC class I, Parainflammation, T-cell co-inhibition and Th2_cells were significantly higher in the patients with subtype2. Similarly, apart from IL6R, TNFSF14, CX3CL1, CX3CR1, and KDR, the remaining immune genes demonstrated high expression in the subtype 2 (Figure 8A). From the perspective of TIME, the wilcoxon signed rank test revealed that patients with sutype2 iCCA presented with higher immune score and estimate score (Figure 8B). Moreover, quanTIseq algorithm determined that monocytes and NK cells were higher infiltrated in the patients with subtype 1 (Figure 8C), TIMER algorithm revealed that subtype2 iCCA presented higher infiltration level of neutrophil and DC cells (Figure 8D), and CIBESORT algorithm showed that T-cell CD4 memory resting was lower infiltrated and macrophages M0 higher infiltrated in the patients with subtype 1 iCCA (Figure 8F). Additionally, the distribution of 19 immune checkpoint genes were not random, and patients with subtype 2 iCCA showed higher expression level of TNFRSF18, TNFRSF9, TNFSF4, IL1A, IL1B, IDO1, CD274, TLR4, IL2RA, PRF1, LAG3, IFNG, TNFSF9, CD70, and ITGB2, and lower expression level of VTCN1, ARG1, EDNRB, and CX3CL1 (Figure 8E). Thus, mitochondrial dysfunction can significantly impact patient’ prognosis and may be associated with alterations in the immune microenvironment.

### Validation of the differential expression of robust MRGs in iCCA

**FIGURE 9:**
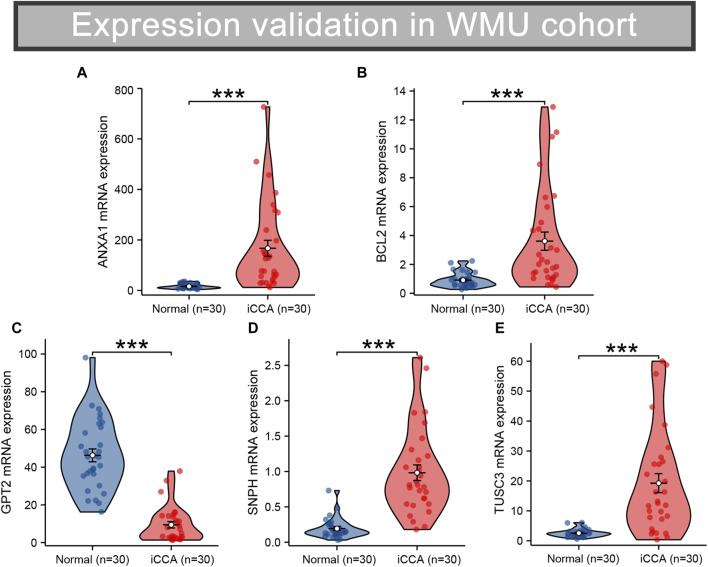
Validation of the differential RNA expression of five robust MRGs in iCCA **(A–E)**. iCCA, Intrahepatic cholangiocarcinoma; MRGs, mitochondria-related genes.

**FIGURE 10:**
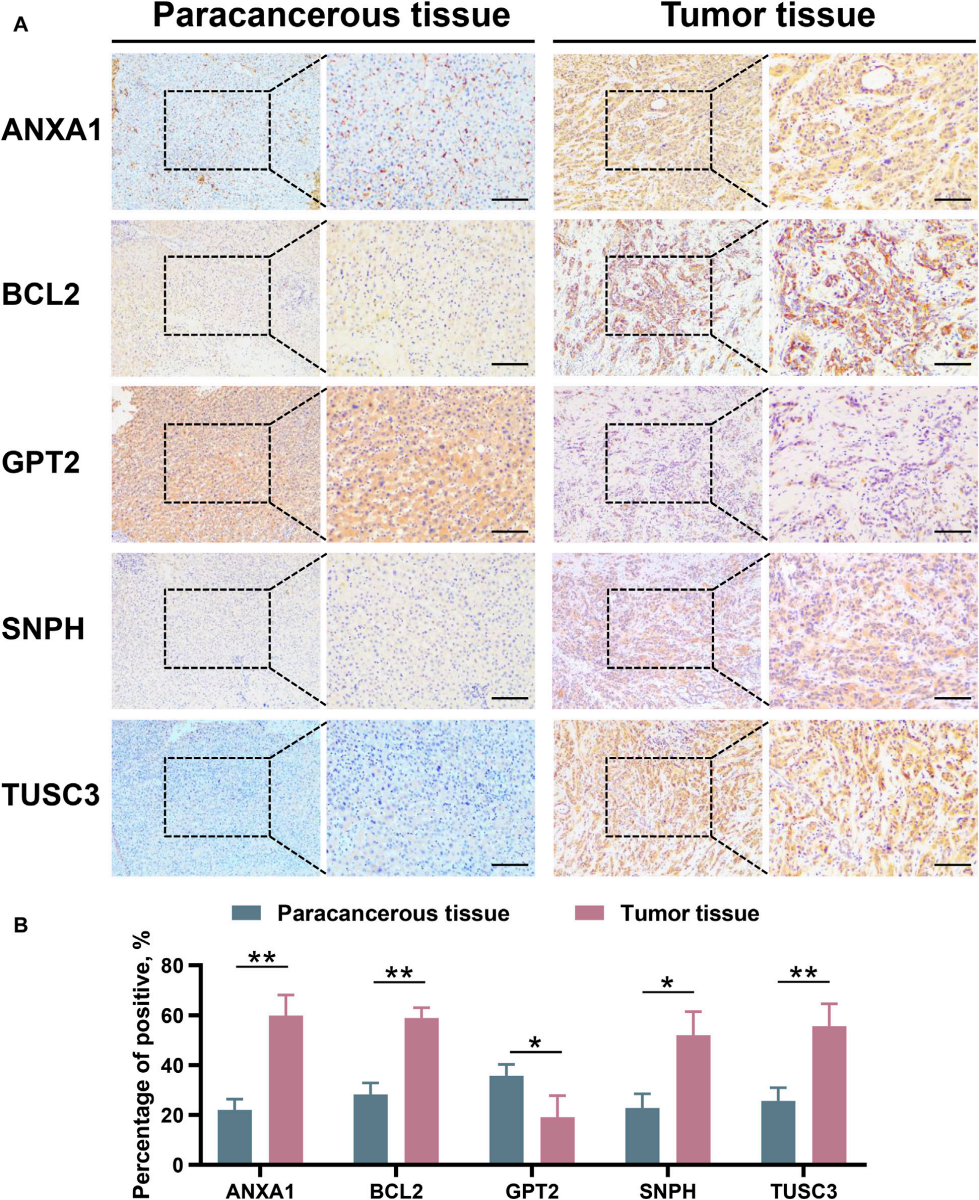
Validation the protein expression level of ANXA1, BCL2, GPT2, SNPH, and TUSC3 between paracancerous and iCCA tissues through IHC. **(A,B)** iCCA, Intrahepatic cholangiocarcinoma; IHC, Immunohistochemistry. *p < 0.05 and **p < 0.01.

RNA sequencing was conducted on 30 pairs of iCCA and normal tissues from the WMU cohort, with the aim of validating the differential mRNA expression levels of five MRGs in the mitochondria signature. As shown in Figures 9A–E, it was observed that ANXA1, BCL2, SNPH, and TUSC3 exhibited significantly elevated expression levels, while GPT2 demonstrated markedly decreased expression in iCCA tissues. Moreover, we collected three matched pairs of cancerous and paracancerous tissues to perform IHC analysis of protein levels for five robust MRGs. We found that the protein expression levels of ANXA1, BCL2, SNPH, and TUSC3 were higher in iCCA tissues compared to paracancer tissues, whereas GPT2 expression was lower in iCCA tissues (Figures 10A, B), consistent with RNA expression levels.

## Discussion

Aberrations and dysfunctions in mitochondrial activity are capable of promoting oncogenesis through a multitude of mechanisms (Warburg, 1956). It has been observed that cancer cells possess the ability to reprogram metabolic patterns by influencing MRGs, thereby creating a favorable environment for their own growth and survival (Roth et al., 2020; Ohshima and Morii, 2021), as exemplified in breast cancer, nasopharyngeal carcinoma, and renal cancer (Wang et al., 2020; Chakraborty et al., 2021; Huang et al., 2021). The association between aberrations in mitochondrial function, as well as perturbations of affiliated genes, and the pathogenesis and aggravation of iCCA remains to be elucidated. Therefore, the aim of this study was to examine the effects of mitochondrial dysfunction on progression, prognosis evaluation, immune microenvironment, and drug sensitivity in iCCA. We constructed a transcriptomic cohort comprising 30 iCCA and adjacent normal tissues, and in conjunction with public TCGA and GSE107943 datasets, interrogated the expression profiles, prognostic associated and functional enrichments of mitochondrial dysfunction associated genes in iCCA. An independent prognostic mitochondria signature has been constructed and externally validated, along with the establishment of mitochondria-associated molecular subtypes, both of which could be utilized for clinical treatment decision-making and prognosis assessment. Mitochondria signature can serve as robust prognostic biomarkers, predicting disease prognosis or treatment response in patients. By correlating specific mitochondrial characteristics with disease progression or treatment response, clinicians can stratify patients into risk groups more effectively and tailor personalized treatment plans. Identifying different mitochondria-associated subtypes may reveal potential therapeutic targets. New treatment strategies can be developed targeting mitochondrial pathways or specific mitochondrial functional impairments associated with different subtypes. This personalized approach can enhance treatment efficacy and reduce adverse reactions. Furthermore, integrating mitochondrial features and subtypes into diagnostic protocols can improve disease classification and patient management, refining disease progression monitoring. In addition, our in-depth exploration revealed crucial insights into how mitochondrial dysfunction influences drug response, modifies the tumor immune microenvironment, and affects immune reactions.

The global incidence of iCCA is progressively increasing. Unfortunately, current screening methods for early-stage detection remain inadequate, thereby contributing to its dismal prognosis (Zhang et al., 2016). Perturbations of mitochondrial genes, encompassing deleterious mutations and aberrant transcription, are closely associated with the development and advancement of various neoplasms, commonly emerging during the initial phase of carcinogenesis (Liu et al., 2022; Huot et al., 2023). Hence, they offer promise for exploitation in early cancer diagnosis. Through interrogation of two datasets, we identified 263 MRGs that were significantly dysregulated in iCCA, encompassing 197 upregulated and 66 downregulated genes. Dysfunction of mitochondrial function leads to disturbances in cellular energy and redox balance, creating favorable conditions for cellular mutations and tumor formation (Zong et al., 2016). In iCCA, we observed that the majority of aberrantly expressed MRGs are associated with mitochondrial part, small molecule metabolic process, mitochondrial envelope, mitochondrial membrane carboxylic, acid metabolic process drug, etc. Notably, KEGG and Hallmarker analyses suggested that these MRGs were typically enriched in amino acid, fatty acid metabolism, and oxidative phosphorylation. These findings highlight the potential impact of MRG malfunction on the balance of acid proportions, thereby disrupting cellular biosynthesis and energy metabolism (Menendez and Lupu, 2007; Solanki et al., 2023). Understanding the intricate interplay between these pathways and mitochondrial dysfunction is crucial for unraveling the underlying mechanisms of iCCA pathogenesis and may open new avenues for therapeutic interventions targeting metabolic dysregulation.

A mitochondria signature with five optimal MRGs was established to evaluate the prognosis of iCCA patients. The AUC values of the signature ranged from 0.785 to 0.928, with a particularly impressive AUC of 0.928 for predicting first-year survival. These findings demonstrate the superior performance of the signature compared to existing prognostic models for iCCA. These five genes have been identified as pivotal regulators of tumorigenesis and progression, exerting their effects through modulation of mitochondrial function, oxidative stress, immune response, apoptosis, and other critical cellular processes. ANXA1 exhibits pro-invasive and pro-tumorigenic properties in multiple cancers, either by triggering autocrine signaling in cancer cells or by shaping a favorable TIME (Araújo et al., 2021). Xiong et al. (2021) showed that RRM2 enhances therapeutic sensitivity to sunitinib and PD-1 blockade in renal cancer through ANXA1 stabilization. Additionally, ANXA1-mediated modulation of Treg cell function impacts patient survival in breast cancer, and therapeutic targeting of ANXA1 attenuates Treg cell function and suppresses breast tumor growth (Bai et al., 2020). BCL2, a well-established oncogene known for its anti-apoptotic properties, has been observed to be overexpressed in various cancer types. In clinical practice, inhibitors targeting BCL2 have shown promising efficacy (Vogler, 2014). Recently, BCL2 was found to participate in mitochondria oxidative stress responses by inhibiting PKM2-mediated apoptosis (Liang et al., 2017). Kim et al. (2019) demonstrates that GPT2 mediates adaptive metabolic responses to glutamine deprivation, suggesting that combining GPT2 and GLS inhibition may be an effective cancer therapy. In addition, GPT2 promotes breast tumorigenesis and cancer stemness via Shh signaling activation, suggesting it is a potential therapeutic target (Cao et al., 2017). Syntaphilin (SNPH) controls proliferation-motility and metastasis decisions via mitochondrial dynamics and rheostat in cancer (Caino et al., 2017). TUSC3 plays a crucial role in promoting drug resistance, cellular stemness, and epithelial-mesenchymal transition (EMT) in tumor progression (Gu et al., 2016). Notably, the differential expression of these five genes has also been validated in the WMU cohort, further supporting their significance in the context of medical research. K-M and ROC curves in the WMU cohort determined the mitochondria signature as a robust and widely applicable prognostic assessment tool.

The TIME of iCCA encompasses a complex immune network that is tumor-dependent. When exposed to innate immune system or drug attack, tumor cells can subtly alter their immune microenvironment, leading to immune tolerance, which can explain observed immunotherapy failures in clinical practice (Moris et al., 2023a). Mitochondria can provide energy and metabolites for immune cells. The generated Ca2+, triglycerides, and active metabolic products can regulate the activation of T-cell and B-cell, thereby playing a role as promoters and regulators (Saha et al., 2022). We found high mitochondria score patients with poor prognosis present lower infiltration levels of T-cell CD4 memory, NK cells, and monocytes. It was possible that the dysfunction of mitochondria inhibits the activation of these immune cells, consistent with findings by other researchers in the context of cancers (O Sullivan et al., 2015; Scharping et al., 2016). Concomitantly, numerous up-regulated immune genes were observed in these patients, which might reflect immune escape mechanisms employed by tumor cells to gain a survival and metastatic advantage (Onkar et al., 2023). Mitochondria are the core of cellular metabolism, and mitochondrial dysfunction can alter the metabolic state of cells (Saha et al., 2022; Onkar et al., 2023; Suomalainen and Nunnari, 2024). This metabolic reprogramming not only creates a high-lactate environment that inhibits effector T-cell and NK cells while promoting the suppressive activity of Tregs, but it also directly restricts the energy supply to immune cells, thereby inhibiting their function and infiltration. The high levels of ROS produced by mitochondrial dysfunction can stimulate tumor cells to release TNF-α, IL-6, and IL-1β, which attract suppressive immune cells and directly damage effector immune cells, reducing their activity. Furthermore, mitochondrial dysfunction can affect multiple signaling pathways, such as HIF-1α and NF-κB, as well as the transmission of communication mediators including cytokines, exosomes, and proteins, thereby influencing the interaction between tumor cells and immune cells.

Furthermore, mitochondrial dysfunction may perturb cellular energetics, affecting drug absorption, transport and metabolism. This can result in drug degradation or accumulation, impacting therapeutic efficacy or toxicity. Meanwhile, some cancer cells alter mitochondrial structure and distribution to reduce drug efficacy through multi-drug resistance mechanisms (Zhang et al., 2023). The mechanisms by which mitochondrial dysfunction influences drug sensitivity are as follows (Rizvi et al., 2018): Mitochondrial dysfunction leads to insufficient energy supply within the cell, which might reduce the sensitivity of tumor cells to energy-intensive treatments such as chemotherapeutic drugs (Sirica et al., 2019); Mitochondrial dysfunction causes an imbalance in intracellular redox reactions, making tumors more resistant to certain oxidative stress-induced therapies (Kilander et al., 2014); Mitochondrial dysfunction results in abnormalities in apoptotic pathways, leading to resistance of tumor cells to apoptosis-inducing drugs (El-Diwany et al., 2019); Mitochondrial dysfunction can affect drug efficacy and resistance through alterations in drug transport and metabolic pathways. The high-risk group exhibited lower IC50 for MG.132, Docetaxel, MS.275, Sorafenib, Sunitinib, and X17.AAG, suggesting that patients with higher mitochondria score may gain advantages from these precision medicines. The modulation of intracellular calcium ion concentration, redox status, and cellular signaling pathways by mitochondrial changes may exert an influence on the targeted oncological response to small molecule drugs.

In addition, we aggregated the WMU, TCGA and GSE107943 cohorts to identify mitochondria-based iCCA subtypes. Two molecular subtypes were determined with significantly different survival outcomes and distinct immune activation and suppression states, further corroborating the link between mitochondrial dysfunction and immune microenvironment, which has significant implications for precision therapy, risk stratification, target discovery, optimization of immunotherapy, and advancement of personalized treatment.

There are some limitations in this study. Firstly, this is a retrospective study, although we conducted validation of the results using the WMU cohort, it is imperative to conduct further investigations and validation of the established mitochondria signature and mitochondria-based iCCA subtypes in larger clinical sample sizes. Secondly, due to limited access to clinical pathological data, this study only included a few clinical factors in a basic multivariate analysis. Future studies should further validate the mitochondria signature as an independent risk factor while comparing its predictive performance against comprehensive clinical indices. Thirdly, the oncogenic roles of key prognostic genes and their interaction mechanisms with mitochondrial dysfunction in iCCA remain unclear and warrant further investigation. Additionally, further biological experiments are necessary to validate the potential mechanisms of mitochondrial dysfunction involved in metabolism and TIME in iCCA. Our future research directions will focus on three main aspects (Rizvi et al., 2018): utilizing cell experiments and mouse models to validate the relationship between the key MRGs we have identified in iCCA, mitochondrial dysfunction, and cancer progression (Sirica et al., 2019); collecting more iCCA cases and clinical data to validate the mitochondria signature, and comparing it with existing models to ensure that the risk model constructed in this study is applicable to clinical practice (Kilander et al., 2014); employing cell experiments combined with transcriptome sequencing to explore the impact of mitochondrial dysfunction on the immune microenvironment and drug sensitivity.

## Conclusion

We found mitochondrial dysfunction modulates aberrant metabolism, oxidative stress, immune responses, apoptosis, and drug sensitivity in iCCA. A mitochondria signature and two mitochondria-based iCCA subtypes were identified for clinical risk stratification and immunophenotyping.

## Data Availability

The datasets presented in this study can be found in online repositories. The names of the repository/repositories and accession number(s) can be found in the article/Supplementary Material.

## Supplementary material

SUPPLEMENTARY FIGURE S1 The workflow of this study.
