# Supplementary figures and images for: Systematic profiling of mitochondria-related transcriptome in tumorigenesis, prognosis, and tumor immune microenvironment of intrahepatic cholangiocarcinoma: a multi-center cohort study
Source: Front Genet. 2024 Jul 26;15:1430885. doi: 10.3389/fgene.2024.1430885 (PMC11310173; doi:10.3389/fgene.2024.1430885)

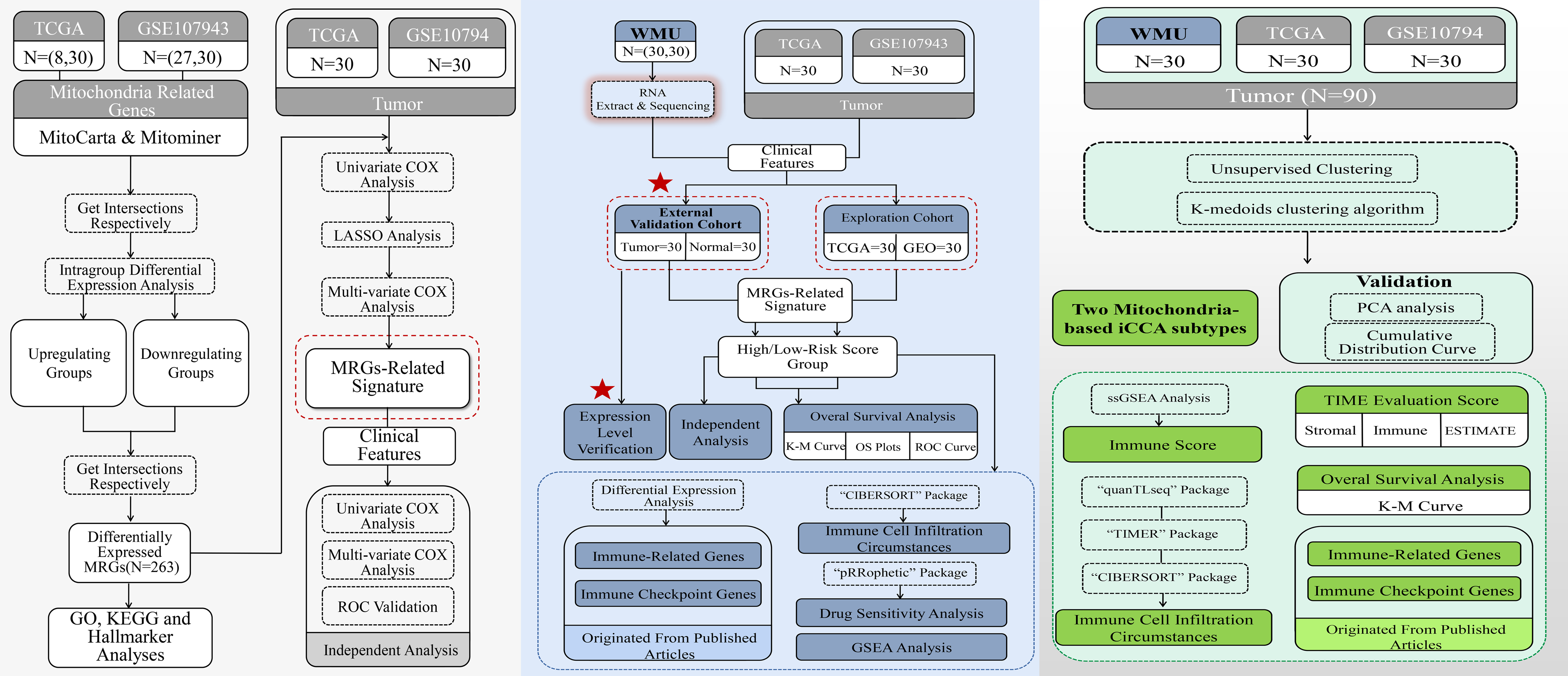

Supplement: Supplementary file 2 [file Image1.TIF]
